# Supplementary material for: A neural command circuit for grooming movement control
Source: eLife. 2015 Sep 7;4:e08758. doi: 10.7554/eLife.08758 (PMC4599031; doi:10.7554/eLife.08758)
Supplement: Supplementary file 1. — Enhancer identities used to target different neuronal classes. DOI: http://dx.doi.org/10.7554/eLife.08758.031 [file elife08758s001.pdf]

Supplementary file 1. Enhancer identities used to target different neuronal classes.

Line Name	Enhancer DBD	Enhancer AD	Enhancer GAL4	Enhancer LexA	Neurons Targeted
aJO-spGAL4-1	R60E02	R52F12			aJO
aJO-spGAL4-2	R25F11	R39A11			aJO
aJO-spGAL4-3	R39A11	R25F11			aJO
aJO-spGAL4-4	R60E02	R39A11			aJO
aJO-spGAL4-5	R39A11	R60E02			aJO
aBN1-spGAL4-1	R34C03	R11B11			aBN1
aBN1-spGAL4-2	R71D01	R11B11			aBN1
aBN1/DN1-spGAL4	R71D01	R34C03			aBN1, aDN1
aBN2-spGAL4-1	R26B12	R24C08			aBN2
aBN2-spGAL4-2	R26B12	R26A02			aBN2
aDN1-spGAL4-1	R71D01	R18C11			aDN1
aDN2-spGAL4-2	R18C11	R76F12			aDN2
aDN2-spGAL4-3	R18C11	R70H02			aDN2
aDN2-spGAL4-4	R76F12	R70H02			aDN2
aJO-GAL4-1			R60E02		aJO
aJO-GAL4-2			R27H08		aJO
aDN-GAL4			R18C11		aDN
aJO-LexA				R27H08	aJO
aBN2-LexA				R26B12	aBN2
aDN-LexA				R18C11	aDN
