# Supplementary material for: A neural command circuit for grooming movement control
Source: eLife. 2015 Sep 7;4:e08758. doi: 10.7554/eLife.08758 (PMC4599031; doi:10.7554/eLife.08758)
Supplement: Supplementary file 2. — Stimulus conditions used for the experiment shown in Figure 6. DOI: http://dx.doi.org/10.7554/eLife.08758.032 [file elife08758s002.pdf]

Supplementary file 2. Stimulus conditions used for the experiment shown in Figure 6.

Tested neuronal pairs	Red light intensity ($\mu W/mm^2$)	Number of pulses delivered	Runs / number of flies tested
aJO to aBN1	~50	50	10 / 3
aJO to aBN2	~50 to 290	10	6 / 3
aJO to aDN1	~ 290 to 800	20	9 / 3
aJO to aDN2	~800	100 to 200	14 / 4
aBN1 to aDN	~50	5 to 20	pre-drug = 13 / 5<br>drug = 24
aBN1 to aBN2	~50	10 to 50	pre-drug = 10 / 3<br>drug = 11
aBN2 to aBN1	~290<br>~800	50<br>20 and 50	pre-drug = 12 / 5<br>drug = 21
aBN2 to aDN1	~290<br>~50	20 to 30<br>20	pre-drug = 13 / 4<br>drug = 19
aBN2 to aDN2	*Mecamylamine*:<br>~ 290<br>~ 500<br>~ 800<br><br>*Picrotoxin*:<br>~50<br>~50<br>~ 290	 15 to 30<br>20<br>50<br><br><br>20<br>50<br>20	 pre-drug = 16 / 5<br>drug = 21<br><br><br><br>pre-drug = 10 / 4<br>drug = 10
pBPLexAp65U control to aBN2	~1000	50	8 / 3
pBPLexAp65U control to aDN2	~800-1000	50	11 / 5
