# Supplementary material for: A neural command circuit for grooming movement control
Source: eLife. 2015 Sep 7;4:e08758. doi: 10.7554/eLife.08758 (PMC4599031; doi:10.7554/eLife.08758)
Supplement: Supplementary file 3. — Stocks used in this study. DOI: http://dx.doi.org/10.7554/eLife.08758.033 [file elife08758s003.pdf]

Supplementary file 3. Stocks used in this study.

Fly stock	References	Figure
w^{1118}; R27H08-LexA (attP40); *5XUAS-DSCP-E86tetLC** (pJFRC34, attP2), *13xlexAop2-IVS-dTrpA1-WPRE*** (JFRC 26, VK0005)	*(Shirangi, Stern, and Truman 2013) **(Mann, Gordon, and Scott 2013)**	**Figure 4B**
*13XLexAop2-IVS-myr-tdTomato*** (pJFRC48, attP18); R27H08-LexA (attP40); *10XUAS-IVS-myr::GFP** (pJFRC12, su(Hw)attP1)	*(Pfeiffer et al. 2010) **(Asahina et al. 2014)	**Figure 5A-D**, **Figure 4 - supplement 2A**,**B**, **Figure 5 - supplement 2A-D**
*13XLexAop2-IVS-myr-tdTomato*** (pJFRC48, attP18); *10XUAS-IVS-myr::GFP** (pJFRC12, su(Hw)attP5); R26B12-LexA (attP2)	*(Pfeiffer et al. 2010) **(Asahina et al. 2014)	**Figure 5E-G**, **Figure 4 - supplement 2C**,**D**, **Figure 5 - supplement 2E-G**
*13XLexAop2-IVS-myr-tdTomato*** (pJFRC48, attP18); *10XUAS-IVS-myr::GFP** (pJFRC12, su(Hw)attP5); R18C11-LexA (attP2)	*(Pfeiffer et al. 2010) **(Asahina et al. 2014)	**Figure 5H**, **Figure 4 - supplement 2E**,**F**, **Figure 5 - supplement 2H**
w^{1118}; R34C03-AD (attP40); *10XUAS-IVS-mCD8::GFP**/TM6b (pJFRC2, su(Hw) attP2)	*(Pfeiffer et al. 2010)	**Figure 2H**
w^{1118}; *10XUAS-IVS-mCD8::GFP* (pJFRC2, attP2)	(Pfeiffer et al. 2010)	**Figure 1C-F**, **Figure 2C-F,H**, **Figure 3A-D**, **Figure 1 - supplement 1B-J**, **Figure 1 - supplement 2A-E'**, **Figure 2 - supplement 1B-H**, **Figure 2 - supplement 2A-I**, **Figure 4 - supplement 1B-D**
w^{1118}; *UAS-dTrpA1* (attP16)	(Hamada et al. 2008)	**Figure 1A-B**, **Figure 2A-B**
w^{1118}; ;*UAS-Kir2.1-GFP*	(Baines et al. 2001)	**Figure 4E** (data not shown)
w^{1118}; *UAS-TeTxLC.TNT*	(Sweeney et al. 1995)	**Figure 4E** **Figure 4 - supplement 3A-C**
20XUAS-CsChrimson-mVenus (attP18)	(Klapoetke et al. 2014)	**Figure 7C-D**, **Figure 6 - supplement 2**, **Figure 6 - supplement 3**, **Figure 7 - supplement 1A**,**B**

*13XLexAop2-CsChrimson-mVenus** (attP18); R24C08-AD (attP40); R26B12-DBD (attP2)	*(Klapoetke et al. 2014)	**Figure 6B**
*13XLexAop2-CsChrimson-mVenus** (attP18); R18C11-AD/CyO (attP40); R71D01-DBD (attp2)	*(Klapoetke et al. 2014)	**Figure 6C**
*13XLexAop2-CsChrimson-mVenus** (attP18); R11B11-AD (attP40); R34C03-DBD (attP2)	*(Klapoetke et al. 2014)	**Figure 6A**
*13XLexAop2-CsChrimson-mVenus** (attP18); R76F12-AD (attP40); R18C11-DBD (attP2)	*(Klapoetke et al. 2014)	**Figure 6D**
*20XUAS-CsChrimson-mVenus** (attP18); *13XLexAop2-IVS-p10-GCaMP6s*** (pGP-JFRC59, su(Hw)attP5); R26B12-LexA/TM6b (attP2)	*(Klapoetke et al. 2014)<br>**(Chen et al. 2013)	**Figure 6F**
w^{1118}; *20XUAS-IVS-GCaMP6s** (pGP-JFRC7) (attP40); R26B12-LexA/TM6b (attP2)	*(Chen et al. 2013)	**Figure 6G-I**
w^{1118}; R27H08-LexA (attP40); *20XUAS-IVS-GCaMP6s**(pGP-JFRC7, VK0005)	*(Chen et al. 2013)	**Figure 6A-D**
20XUAS-CsChrimson-mVenus** (attP18); *13XLexAop2-IVS-p10-GCaMP6s** (pGB-JFRC59, su(Hw)attP5); R18C11-LexA	*(Chen et al. 2013)<br>**(Klapoetke et al. 2014)	**Figure 6E**
w^{1118}; pBDPLexAp65U* (attP40); *20XUAS-IVS-GCaMP6s*** (pGP-JFRC7, VK0005)	*(Pfeiffer et al. 2010)<br>**(Chen et al. 2013)	**Figure 4 - supplement 1A**
w^{1118}; ; pBPGAL4U (attP2)	(Pfeiffer et al. 2010)	**Figure 4B**,**E**<br>**Figure 1 - supplement 1A**,**E**,<br>**Figure 2 - supplement 1A**,<br>**Figure 4 - supplement 3A-C**
w^{1118}; pBPp65ADZpUw* (attP40) ; pBPZpGAL4DBDUw* (attP2)	*(Pfeiffer et al. 2010)	**Figure 1A-B**,<br>**Figure 2A-B**,<br>**Figure 4B**,**E**,<br>**Figure 1 - supplement 1F**,<br>**Figure 5 - supplement 1E**,<br>**Figure 6 - supplement 3** (bottom 2 rows),<br>**Figure 7 - supplement 1A**,**B**
w^{1118}; *lexAop-CD4::spGFP11**, R27H08-LexA/CyO (attP40); *UAS-CD4::spGFP1-10**	*(Gordon and Scott 2009)	**Figure 5 - supplement 1A-H**

pBPhsFlp2::PEST in attP3;; HA_V5_FLAG (MCFO-1)	(Nern, Pfeiffer, and Rubin 2015)	**Figure 1 - supplement 4 B-H**

*/** Asterisks indicate the transgene associated with the corresponding reference in the second column.

References

Asahina, Kenta, Kiichi Watanabe, Brian J Duistermars, Eric Hoopfer, Carlos Roberto González, Eyrún Arna Eyjólfsdóttir, Pietro Perona, and David J Anderson. 2014. "Tachykinin-Expressing Neurons Control Male-Specific Aggressive Arousal in *Drosophila*.." *Cell* 156 (1-2): 221–35. doi:10.1016/j.cell.2013.11.045.

Baines, R A, J P Uhler, A Thompson, S T Sweeney, and M Bate. 2001. "Altered Electrical Properties in *Drosophila* Neurons Developing Without Synaptic Transmission.." *Journal of Neuroscience* 21 (5): 1523–31.

Chen, Tsai-Wen, Trevor J Wardill, Yi Sun, Stefan R Pulver, Sabine L Renninger, Amy Baohan, Eric R Schreiter, et al. 2013. "Ultrasensitive Fluorescent Proteins for Imaging Neuronal Activity." *Nature* 499 (7458): 295–300. doi:10.1038/nature12354.

Gordon, Michael D, and Kristin Scott. 2009. "Motor Control in a *Drosophila* Taste Circuit." *Neuron* 61 (3): 373–84. doi:10.1016/j.neuron.2008.12.033.

Hamada, Fumika N, Mark Rosenzweig, Kyeongjin Kang, Stefan R Pulver, Alfredo Ghezzi, Timothy J Jegla, and Paul A Garrity. 2008. "An Internal Thermal Sensor Controlling Temperature Preference in *Drosophila*." *Nature* 454 (7201): 217–20. doi:10.1038/nature07001.

Klapoetke, Nathan C, Yasunobu Murata, Sung Soo Kim, Stefan R Pulver, Amanda Birdsey-Benson, Yong Ku Cho, Tania K Morimoto, et al. 2014. "Independent Optical Excitation of Distinct Neural Populations." *Nature Methods* 11 (3): 338–46. doi:10.1038/nmeth.2836.

Mann, Kevin, Michael D Gordon, and Kristin Scott. 2013. "A Pair of Interneurons Influences the Choice Between Feeding and Locomotion in Drosophila.." *Neuron* 79 (4): 754–65.

doi:10.1016/j.neuron.2013.06.018.

Nern, Aljoscha, Barret D Pfeiffer, and Gerald M Rubin. 2015. “Optimized Tools for Multicolor Stochastic Labeling Reveal Diverse Stereotyped Cell Arrangements in the Fly Visual System.” *Proceedings of the National Academy of Sciences* 112 (22): E2967–76. doi:10.1073/pnas.1506763112.

Pfeiffer, B D, T-T B Ngo, K L Hibbard, C Murphy, A Jenett, J W Truman, and G M Rubin. 2010. “Refinement of Tools for Targeted Gene Expression in *Drosophila*.” *Genetics* 186 (2): 735–55. doi:10.1534/genetics.110.119917.

Shirangi, Troy R, David L Stern, and James W Truman. 2013. “Motor Control of Drosophila Courtship Song..” *Cell Reports* 5 (3): 678–86. doi:10.1016/j.celrep.2013.09.039.

Sweeney, S T, K Broadie, J Keane, H Niemann, and C J O'Kane. 1995. “Targeted Expression of Tetanus Toxin Light Chain in *Drosophila* Specifically Eliminates Synaptic Transmission and Causes Behavioral Defects.” *Neuron* 14 (2): 341–51.
