# Supplementary material for: A neural command circuit for grooming movement control
Source: eLife. 2015 Sep 7;4:e08758. doi: 10.7554/eLife.08758 (PMC4599031; doi:10.7554/eLife.08758)

Fly Electromagnetic Stimulation

Hampel *et al.* (2015)

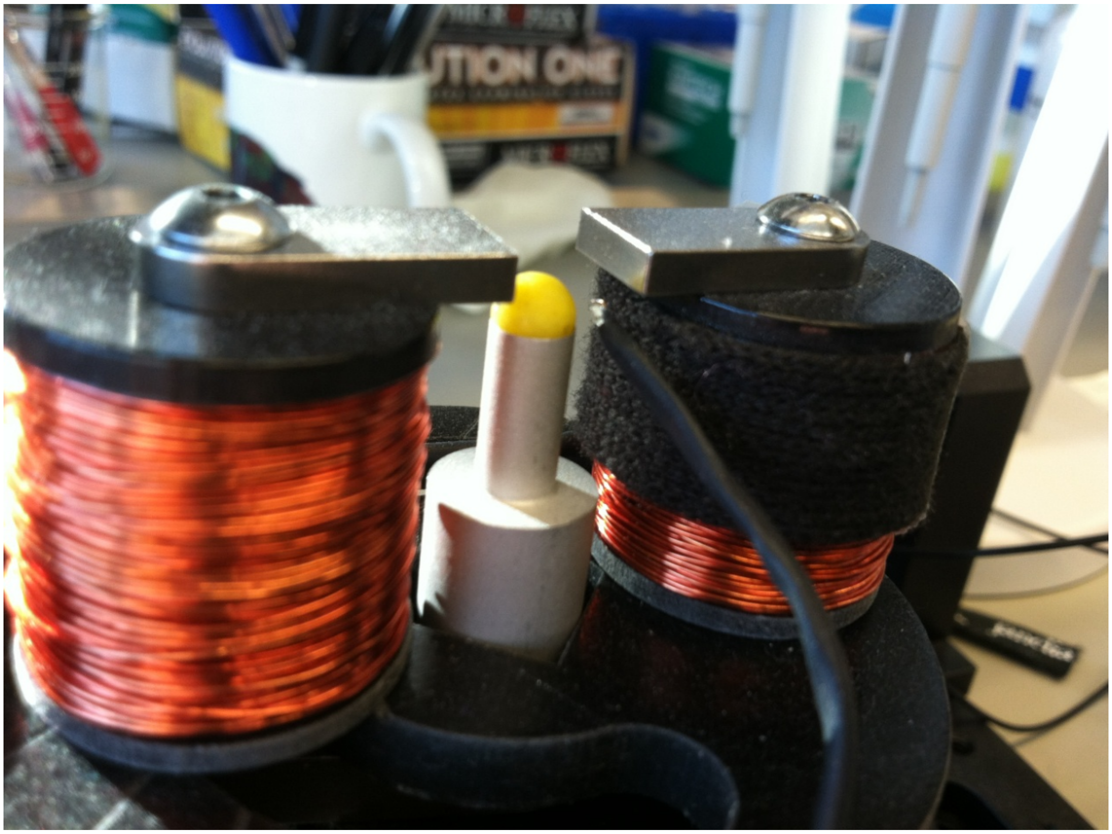

Steven Sawtelle
Electrical Engineer, ID&F
(571) 209-4158

Contents

System Overview 2

Assembly, Test, and Calibration 4

Photographs 4

System Overview

The Fly Electromagnetic Stimulation is designed to provide a strong and controllable magnetic field across a tethered fruit fly in order to stimulate grooming behavior. Fine iron particles are deposited on the fruit fly to provide a place for the magnetic field to act against.

Hardware Development

An electromagnetic was chosen as it could be easily varied in intensity and direction without any physical movement near the fly. A magnetic circuit was designed such that the gap in the field appears across the tethered fly. An IR LED was placed in the camera field to indicate when the field was present for recording purposes. The IR LED drive current is adjustable.

The circuit is controlled by a host computer running a Matlab program, which in turn drives digital outputs on a National Instruments USB-6008 Multifunction I/O board. The digital outputs control the IR LED and drive an isolated solid-state relay. The relay, in turn, controls the connection of power to the electromagnet.

Schematic

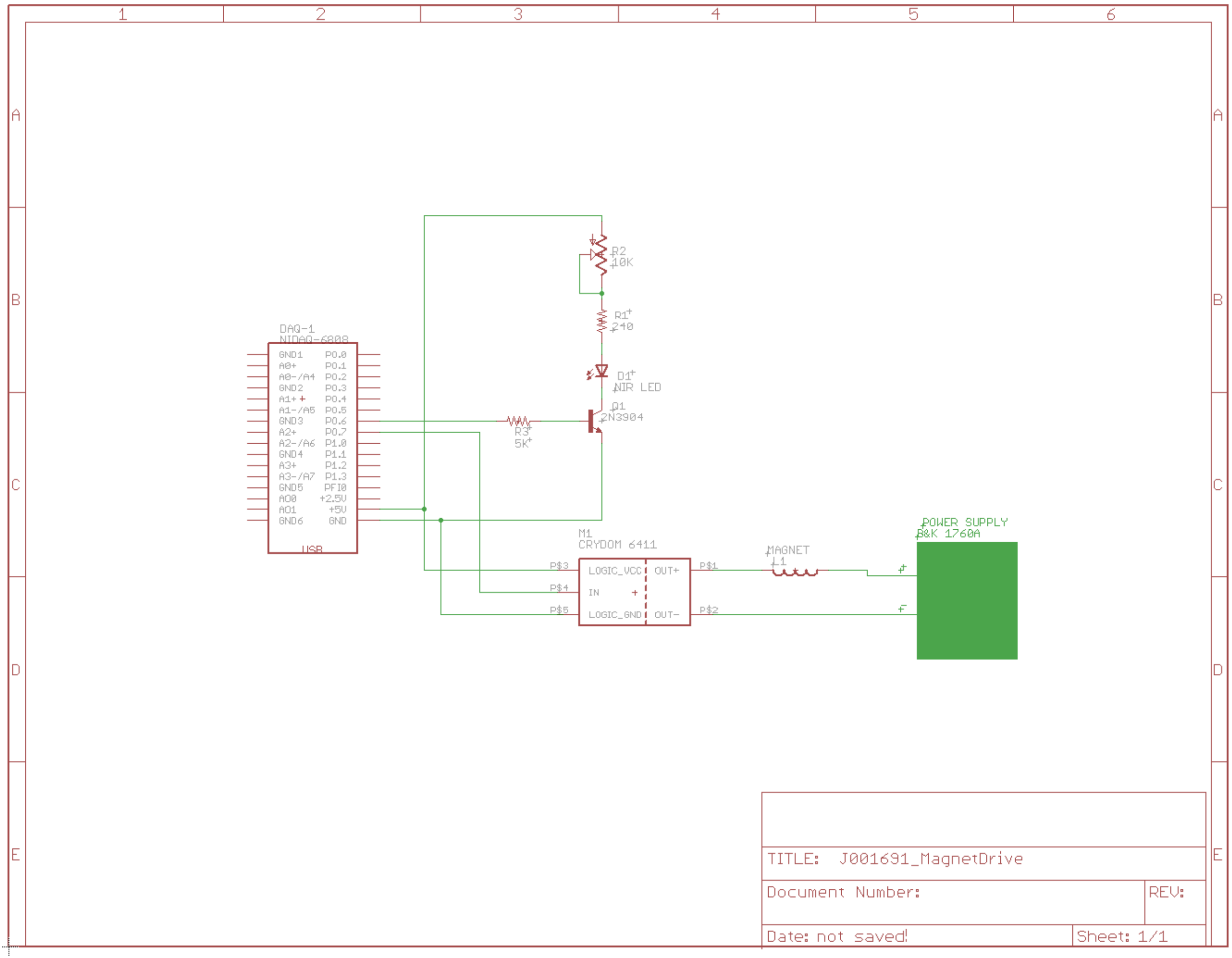

Materials

J001691_MagnetDrive Parts List

Qty	Value	Device	Designators	Manufacturer	PN
1	2N3904	NPN Transistor	Q1	Fairchild	2N3904TA
1	5K	Resistor	R3	Ohmite	OD503JE
1	10K	Potentiometer	R2	Honeywell	308NPC10K
1	240	Resistor	R1	Ohmite	OD241JE
1	6411	Solid State Relay	M1	Crydom	6411
1	(custom)	Magnet	L1	JFRC	
1	USB-6008	NIDAQ	U$1	National Instruments	USB-6008
1	875nm IR	LED	D1	Everlight	SIR19-21C/TR8

HHMI janelia farm research campus

Assembly, Test, and Calibration

The device was assembled on a prototype board. The IR LED brightness is adjusted by a potentiometer and the magnetic field is adjusted by manually varying the power supply current.

Coil construction

Two identical coils were used in the assembly and connected in series. The coils were wound on 3-D printed bobbins 1” tall, 1” diameter, with a 0.365” diameter core. 26AWG magnet wire was wound on each bobbin until it was full; about 700 turns. Resistance per coil was measured to be 6.4 ohms.

Photographs

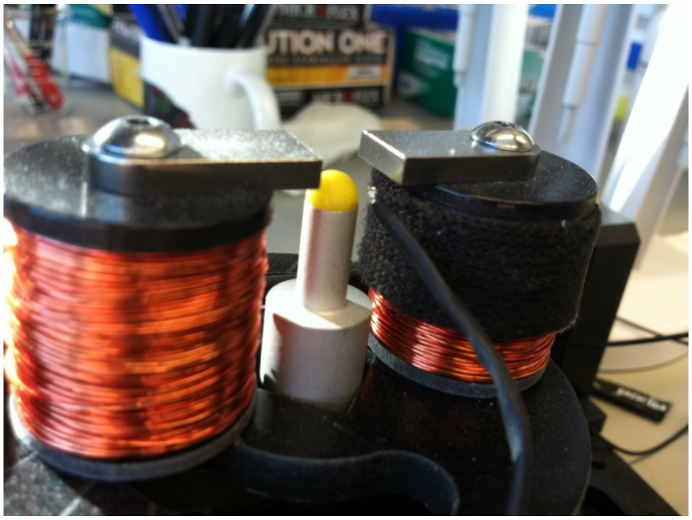

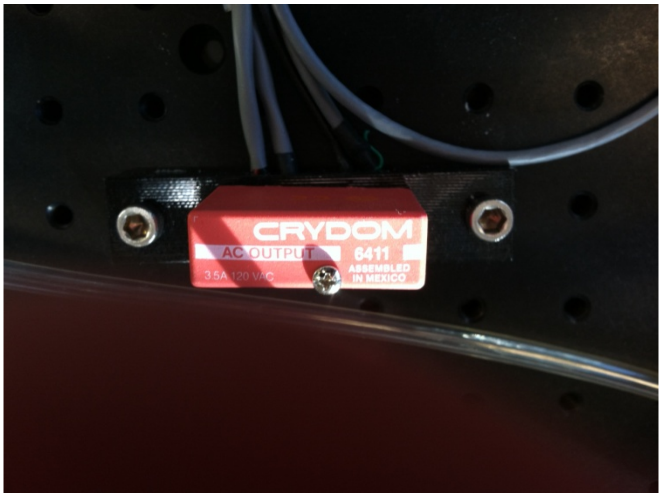
CRYDOM
AC OUTPUT
6411
ASSEMBLED IN MEXICO

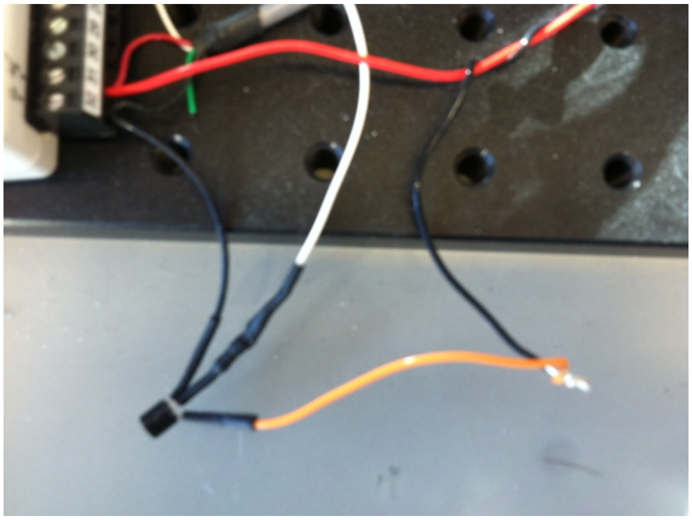

HHMI janelia farm research campus

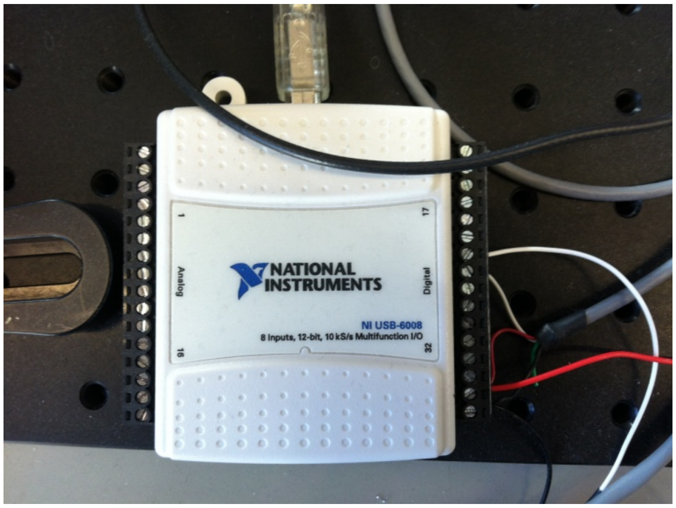

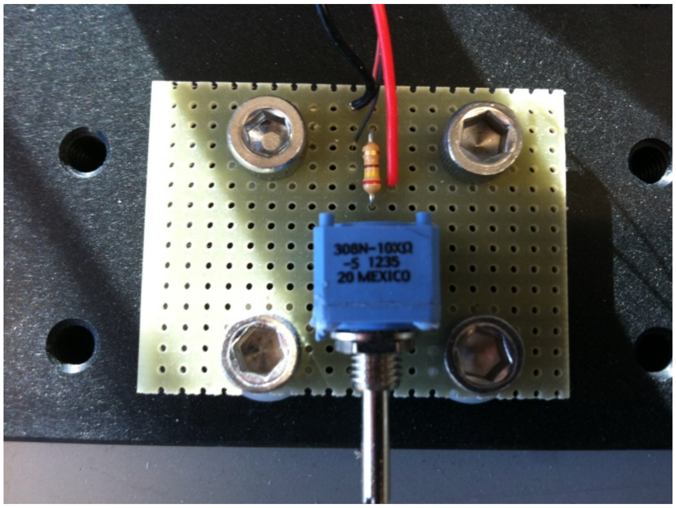

Supplement: Supplementary file 4. — Design plans for the fly electromagnetic stimulation rig. DOI: http://dx.doi.org/10.7554/eLife.08758.034 [file elife08758s004.pdf]
